# Evaluation of one-image 3D reconstruction for plant model generation

**DOI:** 10.1186/s13007-025-01482-6

**Published:** 2026-01-13

**Authors:** Zihe Gao, Zane K. J. Hartley, Andrew P. French

**Affiliations:** School of Computer Science, University of Nottingham, Wollaton Road, Nottingham, NG8 1BB UK

**Keywords:** 3D plant reconstruction, Image-conditioned generation, Diffusion models, Plant digitisation, PlantDreamer dataset, Single-image reconstruction

## Abstract

Generating accurate and visually realistic 3D models of plants from single-view images is crucial yet remains challenging due to plants’ intricate geometry and frequent occlusions. This capability matters because it supplements current plant datasets and enables non-destructive, high-throughput phenotyping for crop breeding and precision agriculture. More broadly, 3D reconstruction is particularly important because plant morphology is inherently three-dimensional, while 2D representations miss occluded leaves, branching geometry, and volumetric traits. However, plants present unique challenges compared to common rigid objects, and most current generative methods have not been systematically tested in this domain, leaving a gap in understanding their reliability for realistic plant reconstruction. This study systematically evaluates six advanced generative techniques–Hunyuan3D 2.0, Trellis (Structured 3D Latents), One2345++, InstantMesh, Direct3D and Unique3D–using the existing PlantDreamer dataset. Specifically, this research reconstructs mesh models from images of Bean plants and quantitatively assesses each method’s performance against ground-truth models using Chamfer Distance, Normal Consistency, F-Score, PSNR, LPIPS, and CLIP Score. The paper also presents qualitative results of Kale and Mint plants. The results indicate that Hunyuan3D 2.0 achieves superior performance overall, suggesting its effectiveness in capturing complex plant structures. This work provides valuable insights into strengths and limitations of contemporary 3D generative approaches, guiding future improvements in realistic plant digitisation.

## Introduction

**Fig. 1:**
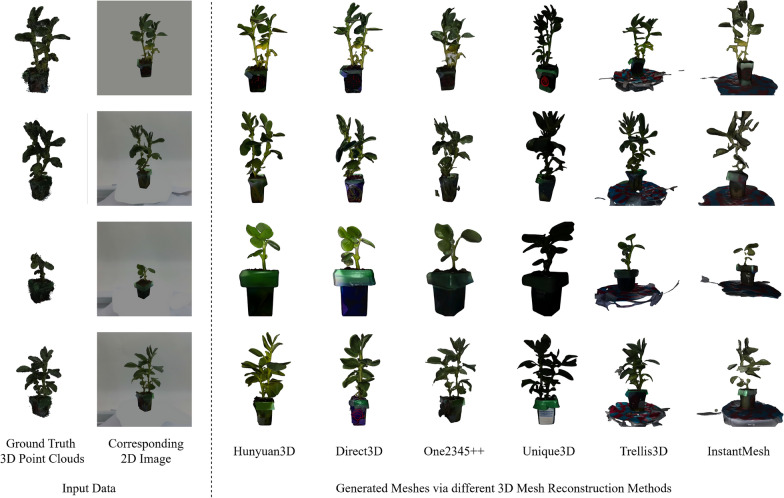
Overview of this evaluation research. On the left are examples of input data, including ground truth 3D plant point clouds and corresponding 2D frontal-view images. The right side shows examples of generated meshes from the given 2D image via different reconstruction methods

The precise and realistic reconstruction of three-dimensional (3D) plant structures from limited-view images has become increasingly critical within plant sciences, impacting research fields such as digital plant phenotyping, crop breeding, ecological monitoring, and precision agriculture. Accurate 3D plant modelling allows researchers and practitioners to non-invasively analyse plant morphology [1, 2], measure phenotypic traits [3, 4], and monitor plant health under various environmental conditions [5, 6], leading to greatly enhanced productivity, sustainability, and resilience in agriculture and ecology [7].

Despite its critical importance, the reliable reconstruction of realistic plant geometries from single or limited-view images remains particularly challenging [8]. Plants, characterised by intricate branching patterns, complex leaf arrangements, thin stems, and high degrees of self-occlusion, represent one of the most structurally challenging classes of objects to reconstruct accurately. Traditional 3D reconstruction approaches, which are originally designed for rigid, artificial objects such as furniture or vehicles, generally fail to handle the inherent structural complexities and irregularities of plants, frequently resulting in incomplete, distorted, or overly simplified geometries [9]. Consequently, specialised approaches tailored explicitly for plants are urgently needed.

### Background

Recent advancements in generative artificial intelligence, particularly image-conditioned generative models, represent a fundamental shift, enabling reconstruction from only a single or a few images. These methods leverage powerful deep-learning techniques, including diffusion models and structured latent spaces, to synthesise realistic 3D structures directly from limited-view imagery [10].

Notably, models like Hunyuan3D 2.0 [11] have introduced sophisticated diffusion pipelines that effectively generate detailed shapes and textures, while Trellis3D [12] leverages structured latent representations (SLAT) to achieve spatial coherence across diverse outputs. Additionally, rapid-generation models such as InstantMesh [13] and One2345++ [14] provide new avenues for real-time or interactive phenotyping applications, though often at the expense of geometric accuracy and detailed fidelity. Emerging approaches such as Unique3D [15] and Direct3D [16] further aim to refine reconstruction consistency and inference speed, yet their suitability and performance in plant-specific contexts require careful evaluation.

Although these generative methods have shown strong performance on standard computer vision benchmarks, results on non-plant datasets do not indicate how well they transfer to plants. Existing evaluations largely rely on rigid or synthetic objects, which lack the intricate branching, thin leaves, and self-occlusions characteristic of real plants. As a result, it remains unclear whether current methods can reliably reconstruct realistic plant structures, highlighting the need for systematic evaluation in this domain [9].

### Motivation and aims of this study

Given the limitations highlighted above, there is an urgent need for comparative evaluations of advanced generative models explicitly focused on realistic plant reconstruction, specifically 3D mesh creation for plants. Most existing evaluations in the literature have primarily targeted rigid, synthetic, or simplified geometries, leaving significant knowledge gaps regarding performance and applicability in botanical scenarios.

The present study aims to directly address this research gap by rigorously evaluating six advanced image-conditioned generative models using the recently developed PlantDreamer dataset [17], which contains high-quality point clouds and corresponding single-view images of diverse plant species (Bean, Kale, Mint). We do this by investigating each method’s capabilities and limitations in accurately capturing the intricate geometries and complex textures inherent in real bean plant structures, as shown in Fig. 1.

The key research objectives are as follows:

1. Conduct a detailed comparative analysis of the selected generative methods (Hunyuan3D 2.0, Trellis3D, One2345++, InstantMesh, Unique3D, and Direct3D) specifically for realistic plant reconstruction.
2. Quantify reconstruction accuracy, visual realism, and semantic consistency using well-established evaluation metrics (Chamfer Distance, Normal Consistency, IoU, F-Score, PSNR, LPIPS, and adapted CLIP Score).
3. Identify practical strengths and limitations of each generative method to inform future development and real-world plant phenotyping applications.

The findings from this study provide critical insights into the capabilities and limitations of existing 3D generative models in realistic plant reconstruction. They also serve as an essential reference for selecting appropriate methods based on application-specific priorities, such as high accuracy, computational efficiency, or real-time performance. Ultimately, the results contribute directly to advancing digital plant phenotyping technologies and pave the way for future methodological innovations tailored explicitly for botanical research and precision agriculture.

While single-image 3D reconstruction holds long-term potential for field phenotyping and agricultural automation, this study focuses specifically on evaluating the technical feasibility of current generative reconstruction models under controlled conditions. The goal is not to propose a ready-to-deploy field solution, but rather to establish an evidence-based understanding of what existing methods can and cannot achieve for plant structures. Practical deployment in production fields will require addressing additional challenges such as variable lighting, background clutter, robustness to occlusion, and biological trait reliability. These aspects are discussed further in the Discussion section.

The remainder of the paper is structured as follows: Section 2 reviews relevant literature, focusing on existing image-conditioned generative techniques, native 3D generative approaches, and plant-specific datasets. Section 3 describes the detailed methodology of this study, including dataset characteristics, experimental workflow, and evaluation metrics. Section 4 presents the experimental results, both quantitatively and qualitatively. In Section 5, this paper discusses the implications of the findings in the context of existing plant reconstruction research, highlighting methodological limitations and practical recommendations. Finally, Section 6 summarises the main conclusions and identifies promising future research directions within plant-specific 3D reconstruction methodologies.

## Related work

### 3D plant reconstruction in plant sciences

In plant science research, accurately capturing plant structure in three dimensions (3D) plays a crucial role in digital phenotyping, crop monitoring, plant growth analysis, and ecological research. Traditional manual measurement methods for plant traits are labour-intensive, subjective, and often destructive [18]. Thus, automated and non-invasive methods of 3D reconstruction from imaging data are gaining increasing importance.

Early plant 3D reconstruction approaches relied on multi-view stereo photogrammetry or laser scanning techniques (e.g., terrestrial laser scanning or LiDAR [9]). These methods produce highly accurate and detailed 3D representations but typically require extensive equipment setup, controlled conditions, multiple viewpoints, and considerable manual processing [18]. More importantly, the complex structural characteristics of plants–such as thin, delicate leaves, overlapping branches, and severe occlusions–often present challenges for traditional photogrammetric or laser-based techniques, frequently resulting in incomplete or distorted reconstructions. Any multi-view or scanning approach can also be hampered by the plant moving in-between captures, which would be a common problem in an outside environment.

Recent developments in computer vision, especially deep-learning-based generative models, have shown significant potential to address these challenges (for example, RGB cameras are more cost-effective and easier to access [19]). Image-conditioned generative methods that use powerful deep neural networks trained on large-scale datasets promise automated, scalable, and accurate reconstructions directly from limited or even *single-view* imagery, offering new opportunities for precision agriculture and automated phenotyping applications.

### Image-conditioned 3D generation methods via 2D priors

Image-conditioned 3D generation methods via 2D priors leverage strong prior knowledge learned from large-scale image datasets to predict accurate and realistic 3D structures from limited-view images. Early approaches typically employed explicit geometric representations such as voxels [19] or point clouds [20]. However, recent advances have favoured implicit neural representations and diffusion-based generative approaches due to their superior detail fidelity and flexibility [21].

DreamFusion [22] marked a significant advancement in this area by introducing Score Distillation Sampling (SDS) [22], a technique where a pre-trained text-to-image diffusion model is used to optimise a 3D representation so that its renders are scored highly by the diffusion model for a given prompt. In this case, the 3D representation is a Neural Radiance Field (NeRF) [23], an implicit model that maps 3D coordinates and viewing directions to colour and density, allowing objects or scenes to be represented as continuous volumetric fields that can be rendered from novel views. While originally demonstrated in text-to-3D tasks, the underlying principles of SDS are applicable to single-image-to-3D reconstruction, enabling the generation of detailed textures and realistic geometries.

Zero123 [24] further improved multi-view consistency by synthesising additional viewpoints from a single input image, enhancing reconstruction accuracy across multiple perspectives. Similarly, One2345 [25] and its improved variant One2345++ [14] adopted a two-step pipeline–first synthesising multiple views, then performing implicit surface reconstruction via diffusion methods–to provide rapid, though sometimes less precise, reconstructions suitable for interactive applications. However, evaluations specifically targeting plant reconstruction scenarios remain limited, and performance typically declines with increasing geometric complexity, such as dense foliage or intricate branching patterns.

### Native 3D generative methods

In contrast to image-conditioned models that heavily rely on 2D priors, native 3D generative methods directly operate in a dedicated 3D representation space. Recently developed models, including Hunyuan3D 2.0 [11] and Trellis3D [12], represent important advancements in native 3D generative approaches, demonstrating improved reconstruction quality and versatility.

Hunyuan3D 2.0 employs a sophisticated two-stage pipeline involving diffusion transformer-based geometry generation and subsequent high-resolution texture synthesis via diffusion painting. This approach has proven particularly effective for producing detailed and realistic reconstructions of complex geometries and textures, showing potential for intricate botanical structures. The implementation details of different pipelines mentioned in this section are further explained in Sect. 3.3.

Trellis3D introduced Structured 3D Latents (SLAT), enabling flexible decoding into multiple 3D output formats (meshes, NeRFs, Gaussian splats [21]). Its structured latent representation significantly improves spatial coherence and flexibility, offering advantages in diverse plant modelling contexts.

Rapid generation methods, such as InstantMesh [13], achieve remarkable inference speeds through differentiable iso-surface extraction from learned implicit representations. These methods are especially promising for real-time interactive phenotyping applications, though often at the cost of geometric precision and fidelity.

Newer native methods, such as Unique3D [15] and Direct3D [16], further refine implicit-based reconstructions and direct mesh generation methods, respectively, targeting improved multi-view consistency and computational efficiency. However, comprehensive plant-focused evaluations for these emerging methods remain scarce, motivating the necessity of rigorous comparative assessments specifically tailored to realistic plant scenarios.

### Available plant-specific datasets

The availability of suitable datasets remains a critical limitation for realistic plant modelling research. Traditional 3D datasets, such as ShapeNet [26] or ModelNet [27], primarily comprise synthetic objects or rigid man-made structures, failing to represent plant complexity accurately. Datasets specifically developed for plant phenotyping, such as PhenoBench [28] and CVPPP datasets [29], often focus on segmentation or classification tasks rather than full-plant 3D reconstruction.

Recently, specialised datasets such as PlantNet [30] and PlantDreamer [17] have emerged, offering accurate and precise point clouds of real plants accompanied by corresponding image data. Notably, the PlantDreamer dataset, developed at the University of Nottingham, provides carefully acquired and controlled point-cloud reconstruction paired with single-view images of various plant species, including Bean, Kale, and Mint. This dataset represents a significant advancement, enabling precise quantitative and qualitative assessments of 3D generative models for plant reconstruction tasks.

**Fig. 2:**
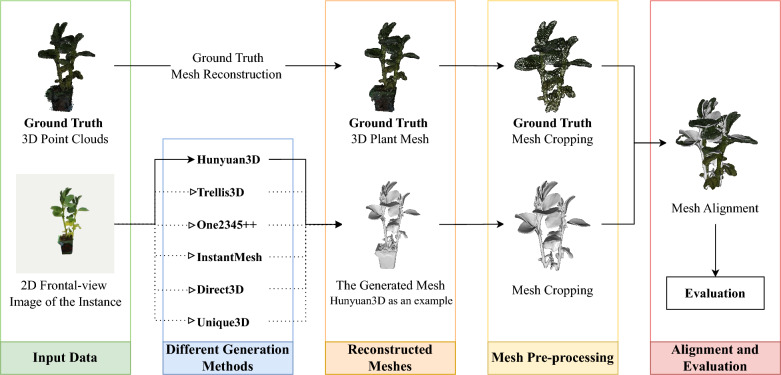
Experimental pipeline for 3D plant model generation and evaluation. The top path represents the Ground Truth data. The original 3D plant point cloud is first reconstructed into a mesh representation, followed by standard pre-processing steps (cropping and alignment) to provide a consistent reference for evaluation. The bottom path illustrates the generation pipeline. A 2D frontal-view image of the same plant instance is given as input to different 3D generation methods. These methods output 3D plant meshes, which are subsequently reconstructed, cropped, and aligned to ensure comparability. Finally, the generated meshes are quantitatively and qualitatively evaluated against the processed Ground Truth mesh. For clarity, the figure shows Hunyuan3D as an example in the evaluation stage

## Methods

### Aim, design, and experimental workflow

The primary aim of this study was to systematically evaluate the performance of several state-of-the-art image-conditioned 3D generative models specifically in the context of realistic plant reconstruction from single-view images. To achieve this aim, this research conducted a comparative analysis involving six advanced generative models: Hunyuan3D 2.0 [11], Trellis3D (Structured 3D Latents, SLAT) [12], One2345++ [14], InstantMesh [13], Unique3D [15], and Direct3D [16]. These models were selected based on their prominence, diversity of underlying methodologies, and practical suitability for plant reconstruction tasks.

The experimental workflow consisted of four main stages shown as Fig 2: ground truth model and image preparation, generative model inference, model pre-processing and alignment, and quantitative and qualitative evaluations. Each stage will be introduced in the following sections of this chapter.

### Dataset characteristics and plant material

**Fig. 3:**
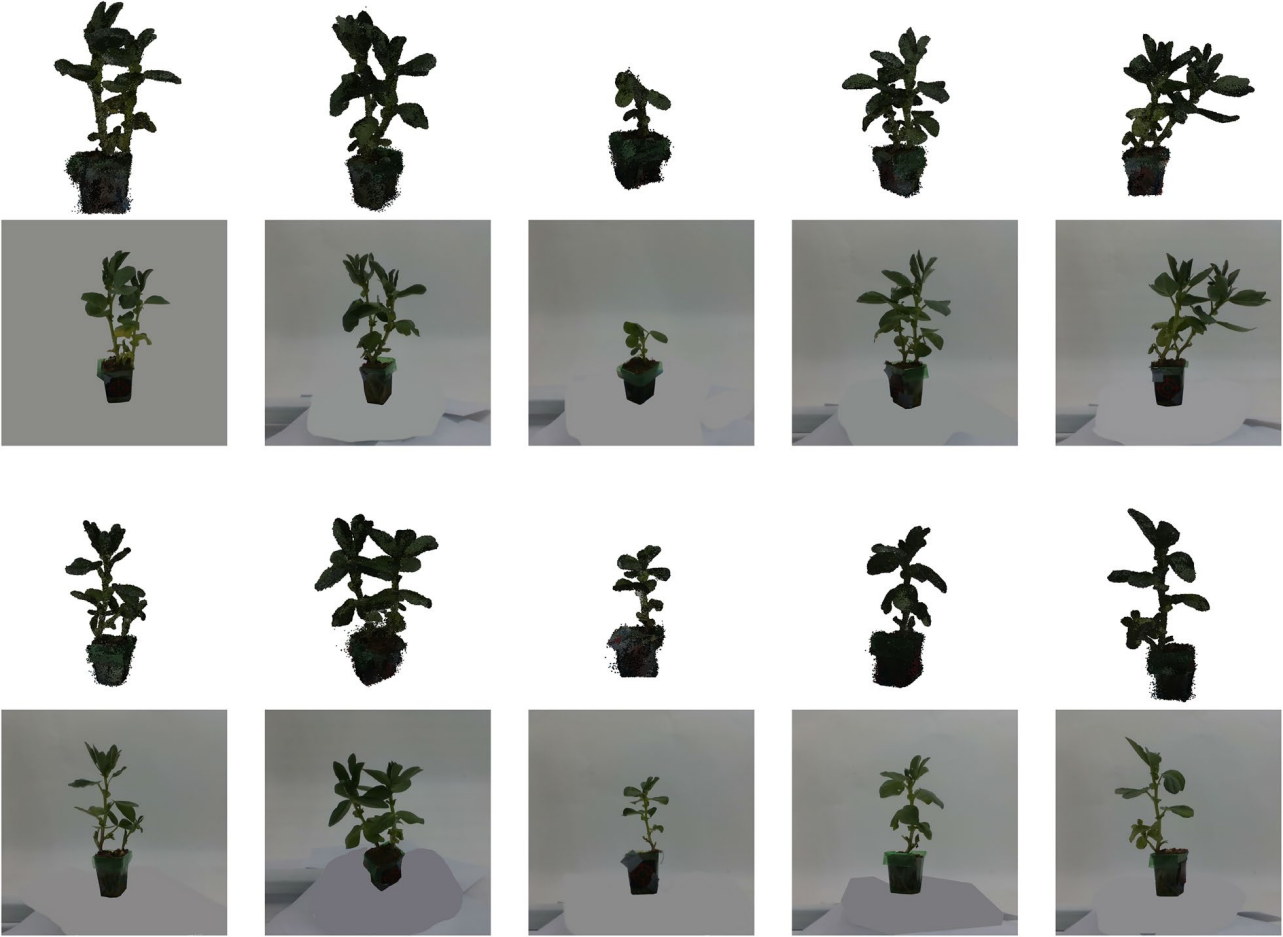
10 individual Bean plant instances from the PlantDreamer dataset are selected as Ground Truth data, and their corresponding 2D frontal-view images are input data for 3D mesh reconstruction process

This study used the PlantDreamer [17] dataset, a specialised dataset recently developed by researchers at the University of Nottingham, explicitly aimed at facilitating rigorous quantitative and qualitative assessments of 3D plant reconstruction methods. The PlantDreamer dataset includes accurate 3D reconstruction provided as dense point-cloud models, complemented by extensive multi-view RGB image data. Specifically, for each individual plant instance, the dataset provides over one hundred RGB images captured from multiple viewpoints around the plant, along with corresponding segmented versions of these images (with the background removed), and specially processed images where the base under the pot is digitally painted out.

In this experimental protocol, ten individual Bean plant instances from the dataset were arbitrarily selected as shown in Fig 3, considering their clear structural complexity, like clear stems and leaves, and intermediate level of geometric detail, which provided a suitable basis for systematic comparison. For each Bean plant instance, these experiments selected the single-view RGB image captured from the frontal viewpoint of the plant as the standard input for subsequent 3D reconstruction processes. Since some methods (Trellis3D and InstantMesh) to be evaluated lacked robust, integrated image-segmentation or background-removal capabilities, for these two methods, this research substituted the original RGB images with corresponding segmented images (background removed) to ensure optimal reconstruction results and fair comparisons across models, as shown in Fig. 1. To get the fairest results of evaluation of different methods, the final evaluation process is conducted after cropping pots and bases of the meshes, and only generated meshes of plants themselves are kept for comparison as shown in Fig. 2.

To prepare ground-truth reference models for accurate evaluation, the original 3D point-cloud data provided by PlantDreamer was converted into precise ground-truth mesh models using the Alpha Shapes reconstruction algorithm [31]. This method effectively preserves detailed morphological features of the plants, ensuring an accurate representation suitable for rigorous comparative analyses. Other plant models, such as 6 mint models and 3 kale models from PlantDreamer dataset, are also generated for qualitative evaluation.

### Evaluated generative methods and computational platforms

This research evaluated six state-of-the-art generative methods, each chosen for its innovative approach and distinct strengths in reconstructing 3D geometry and textures from single-view images.

Hunyuan3D 2.0 [11] employs a sophisticated two-stage generative pipeline. Initially, geometry is generated through a diffusion transformer network specifically optimised for capturing detailed structural information. Subsequently, a high-resolution diffusion-based painting stage produces realistic surface textures, enhancing visual realism and accuracy. Hunyuan3D 2.0 is particularly noted for its detailed output quality, making it highly promising for reconstructing intricate plant geometries.

Trellis3D [12] introduces Structured 3D Latents (SLAT), leveraging a unified sparse structured latent representation capable of decoding into multiple versatile output formats, including meshes, neural radiance fields (NeRFs), and Gaussian splats. Trellis3D’s latent structure supports consistent spatial reconstruction, positioning it as a versatile choice for complex botanical structures.

One2345++ [14] uses an implicit neural representation based on signed distance functions (SDFs), optimized for rapid, single-step inference. Despite its high computational efficiency, preliminary evaluations have indicated limitations in accurately reconstructing complex plant geometries, especially those with intricate branching or high levels of occlusion.

InstantMesh [13] represents an ultra-fast reconstruction method emphasising real-time interactive performance. It combines differentiable iso-surface extraction techniques with learned implicit representations, resulting in rapid generation suitable for immediate visualisation. However, previous evaluations [12] have noted reduced geometric precision compared to slower, more computationally intensive methods.

In addition, this paper included preliminary evaluations of two newly proposed methods, Unique3D [15] and Direct3D [16]. Unique3D employs advanced implicit neural fields designed specifically to enhance multi-view consistency and spatial accuracy. Direct3D adopts a direct mesh inference approach optimised explicitly for computational speed, robustness, and efficient geometry extraction. These two models were evaluated to identify their potential contributions and suitability for plant-specific reconstruction tasks, complementing the assessment of previously established generative techniques.

Due to the high computational resource demands, particularly GPU memory requirements, associated with contemporary 3D generative models, this research leveraged cloud-based demo platforms publicly available online for conducting these experiments. These platforms allowed direct use of pre-deployed models, thus eliminating the need for extensive local computational infrastructure. For Hunyuan3D 2.0, Tencent’s official Hunyuan3D demo platform was used, accessible through the Tencent Hunyuan3D website [32]. Trellis3D reconstructions were performed via the publicly-accessible Hugging Face Space provided by the Trellis community [33]. The One2345++ method was accessed through the SudoAI platform [34]. InstantMesh reconstructions were generated using the official InstantMesh Hugging Face Space provided by TencentARC [35]. For Direct3D evaluations, the Neural4D official online studio was used [36], and Unique3D reconstructions were conducted through the AIUni platform [37].

Each Bean plant instance image (only 1 original RGB or segmented image, depending on the method) was individually uploaded and processed on the appropriate computational platform to produce corresponding reconstructed 3D meshes. The models generated from these platforms were then downloaded in standard mesh formats (typically GLB) for subsequent local processing and analysis. As to the inference time of different generative methods, it could be affected by the optimisation of different platforms or even the stability of the website because we used cloud-based platforms. But in general, a 3D mesh is generated from one given image in less than 2 min for all selected methods.

### Pre-processing and alignment of generated and ground-truth models

Upon generating the 3D models from the single-view input images, additional pre-processing steps were undertaken to ensure fair and accurate evaluation comparisons with the ground-truth meshes. Specifically, the pot and base components of both ground-truth and generated meshes were cropped out, leaving only the plant structures themselves. This cropping step was essential to eliminate extraneous variables, such as pot geometry and base supports, which are unrelated to the plant reconstruction quality assessment.

A crucial pre-processing step performed prior to the quantitative evaluation involved the accurate spatial alignment of each pair of corresponding generated and ground-truth meshes. Non-alignment would mean the metrics used to calculate the distance between the meshes would be unreliable. Model alignment is an inherently challenging task in 3D reconstruction studies, particularly when the structural similarity between the generated and ground-truth models is moderate or low. Due to these challenges, this research employed a semi-automated alignment procedure involving initial approximate alignment based on automatically computed centroids and bounding-box orientations for scaling, followed by careful manual refinement and the Iterative Closest Point (ICP) algorithm [38]. ICP is a widely used algorithm for rigid registration, which iteratively refines the alignment of two 3D shapes by minimising the distance between corresponding points on their surfaces. This combination was adopted to achieve as precise spatial correspondence as possible for each pair of meshes. The details, challenges, and implications of the alignment procedure are discussed further in the Discussion chapter 5.4 of this paper, highlighting alignment as an important topic for future methodological development.

### Quantitative evaluation metrics

To systematically evaluate and compare model performance, here we selected established quantitative metrics commonly employed in 3D reconstruction research, ensuring comprehensive coverage of different facets of what makes a model "good": geometric accuracy, visual fidelity, and semantic consistency.

For *geometric accuracy* evaluation, we computed the Chamfer Distance, Normal Consistency, IoU and F-Score. Chamfer Distance [39] measures the average nearest-neighbour distance between points on the generated mesh and ground truth, with lower values indicating better geometric accuracy. Normal Consistency [40] quantifies how accurately generated surface normals align with those of the ground truth, where higher values indicate better reconstruction quality. The F-Score [41] combines completeness and precision of reconstructed meshes based on a predefined threshold distance (set at 1% of the bounding box diagonal), with higher scores signifying more accurate and complete meshes.

To assess *visual fidelity*, we used Peak Signal-to-Noise Ratio (PSNR) and Learned Perceptual Image Patch Similarity (LPIPS). PSNR [42] provides a pixel-level similarity measure between rendered images of the generated and ground-truth meshes; higher PSNR values reflect superior visual similarity. LPIPS [43] quantifies perceptual visual similarity from the perspective of human observers, with lower scores indicating higher perceived realism and image quality.

To measure *semantic consistency* between generated and ground-truth renderings, we used an adapted form of the Contrastive Language-Image Pre-training (CLIP) Score [44]. Unlike the conventional CLIP Score typically used for evaluating similarity between images and textual captions, this research modified this metric to compute the cosine similarity between image embeddings directly derived from the ground-truth and generated model renderings. This adapted CLIP Score thus quantified the semantic alignment and perceptual similarity specifically between two corresponding images.

### Statistical analysis and computational details

Quantitative metrics were calculated individually for each Bean plant instance across all six evaluated generative methods. We then computed mean values and standard deviations for each metric across the entire set of ten Bean plant samples per model.

All mesh cropping, scaling, alignment, rendering, and metric computations were conducted locally using standardised computational packages and Python libraries (e.g., Open3D V0.19.0, Trimesh V4.6.4, NumPy V2.1.3, SciPy V1.15.2). Mesh rendering was performed using consistent camera viewpoints and standardised lighting conditions to ensure comparability. Finally, all qualitative analyses and graphical visualisations were generated in Python, ensuring a clear and reproducible presentation of experimental results.

## Results

### Quantitative evaluation results

**Table 1:** Result table of geometry metrics for all methods. Mean of 10 instances, St. Dev. in brackets

	Geometry Metrics			
Methods	CD $\downarrow$	NC $\uparrow$	IoU $\uparrow$	F-Score $\uparrow$
Hunyuan3D	**0.00515 (0.000934)**	*0.3691 (0.0511)*	**0.07212 (0.0322)**	**0.8597 (0.0508)**
Trellis	0.00626 (0.000877)	0.3351 (0.0254)	0.06534 (0.0249)	0.7920 (0.0506)
One2345++	0.00593 (0.001170)	0.3481 (0.0674)	0.04785 (0.0116)	0.8059 (0.0683)
InstantMesh	0.00795 (0.002250)	**0.3715 (0.0980)**	0.04181 (0.0183)	0.7040 (0.1144)
Direct3D	*0.00562 (0.001104)*	0.3646 (0.0562)	*0.07074 (0.0188)*	*0.8236 (0.0706)*
Unique3D	0.00603 (0.001337)	0.3228 (0.0361)	0.06086 (0.0256)	0.8129 (0.0626)

CD for Chamfer Distance, NC for Normal Consistency and IoU for Intersection over Union. The highest scores for each metric are in bold and the second-highest are in italic

**Table 2:** Result table of Visual Fidelity and Semantic Consistency for all methods. Mean of 10 instances, St. Dev. in brackets

	Visual Fidelity		Semantic Consistency
Methods	PSNR $\uparrow$	LPIPS $\downarrow$	CLIP $\uparrow$
Hunyuan3D	6.4972 (0.3048)	0.4469 (0.0618)	**0.8564 (0.0213)**
Trellis	**6.7710 (0.4638)**	0.4722 (0.0536)	0.7886 (0.0577)
One2345++	*6.5442 (0.2289)*	*0.4409 (0.0736)*	0.8288 (0.0567)
InstantMesh	6.4478 (0.2924)	0.4768 (0.0593)	0.7394 (0.0704)
Direct3D	6.5166 (0.3039)	**0.4359 (0.0655)**	*0.8305 (0.0342)*
Unique3D	6.3589 (0.2927)	0.4531 (0.0674)	0.7869 (0.0695)

The highest scores for each metric are in bold and the second-highest are in italic

**Fig. 4:**
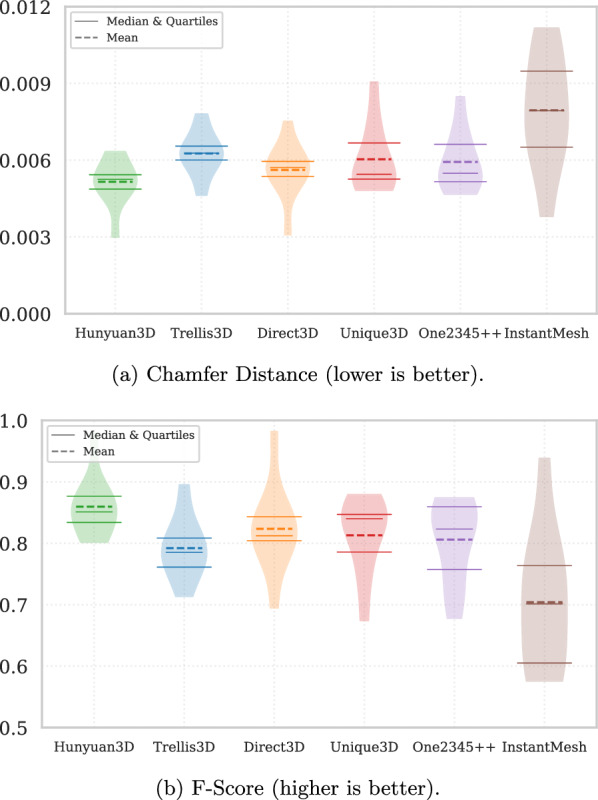
Distribution of quantitative geometry metrics across methods. **a** Chamfer Distance (lower is better). **b** F-Score (higher is better). Each violin plot shows the distribution across 10 Bean instances, with the central dashed line indicating the mean and solid lines indicating the quartiles and median

The quantitative evaluation results for the six advanced image-conditioned generative models–Hunyuan3D 2.0, Trellis3D (Structured 3D Latents, SLAT), One2345++, InstantMesh, Direct3D, and Unique3D–are summarised in Tables 1 and 2, based on ten Bean plant instances from the PlantDreamer dataset. Geometry-focused metrics (Table 1) capture surface accuracy and volumetric completeness, while appearance- and semantics-oriented measures (Table 2) reflect visual realism and how well the generated meshes correspond to the conditioning images. Standard deviations are also reported to indicate the stability of each method across plant instances.

In addition to the mean and standard deviation values reported in Table 1 and 2, Fig. 4 illustrates the full distribution of Chamfer Distance and F-Score across 10 Bean instances for each method. These violin plots highlight both the central tendency and variability, showing that Hunyuan3D consistently achieves the lowest Chamfer Distance and highest F-Score, whereas InstantMesh exhibits larger variance and generally weaker performance.

In terms of geometric accuracy, Hunyuan3D demonstrated the best overall performance, achieving the lowest Chamfer Distance (0.00515 ± 0.00093) and the highest F-Score (0.8597 ± 0.0508), indicating excellent overall geometric precision and completeness. Its Normal Consistency (0.3691 ± 0.0511) was slightly lower than InstantMesh, but still a strong second place among all evaluated methods. Additionally, Hunyuan3D attained the highest semantic alignment with the adapted CLIP Score (0.8564 ± 0.0213), underscoring its superior semantic consistency with the input image content.

*Direct3D* also exhibited strong geometric performance, achieving the second-lowest Chamfer Distance (0.00562 ± 0.00110), accompanied by robust Normal Consistency (0.3646 ± 0.0562) and IoU (0.07074 ± 0.0188), indicating its effectiveness in capturing spatial structures. Its semantic consistency (CLIP Score: 0.8305 ± 0.0342) and visual fidelity (LPIPS: 0.4359 ± 0.0655, PSNR: 6.5166 ± 0.3039) also placed it among the top-performing methods in this evaluation. It is important to note that the PlantDreamer dataset does not provide physical scale for the original point clouds, and the reconstructed meshes are normalised during alignment; therefore, the Chamfer Distance values should be interpreted only in a relative sense.

Beyond Hunyuan3D 2.0 and Direct3D, the remaining four methods showed mixed and generally more limited performance. One2345++ achieved reasonable geometry overall but consistently struggled with volumetric completeness, leading to lower IoU scores. It nevertheless produced perceptually sharper renders, as indicated by its strong performance on LPIPS, making it visually convincing despite underlying structural errors. Trellis3D offered balanced but unspectacular results: while its geometric measures were only moderate, it stood out for delivering the highest PSNR values, suggesting that at the pixel level its renders aligned well with the ground truth, albeit with reduced perceptual realism. InstantMesh was the fastest method evaluated and excelled in producing smooth, well-aligned surfaces (reflected in high normal consistency), but these came at the expense of accurate volumetric reconstruction and fine structural detail, limiting its usefulness for quantitative plant analysis. Finally, Unique3D produced coherent global plant shapes and acceptable geometry but tended to underperform in visual and semantic alignment compared to the stronger methods, placing it in the lower-middle tier of overall performance.

### Qualitative visual comparisons

**Fig. 5:**
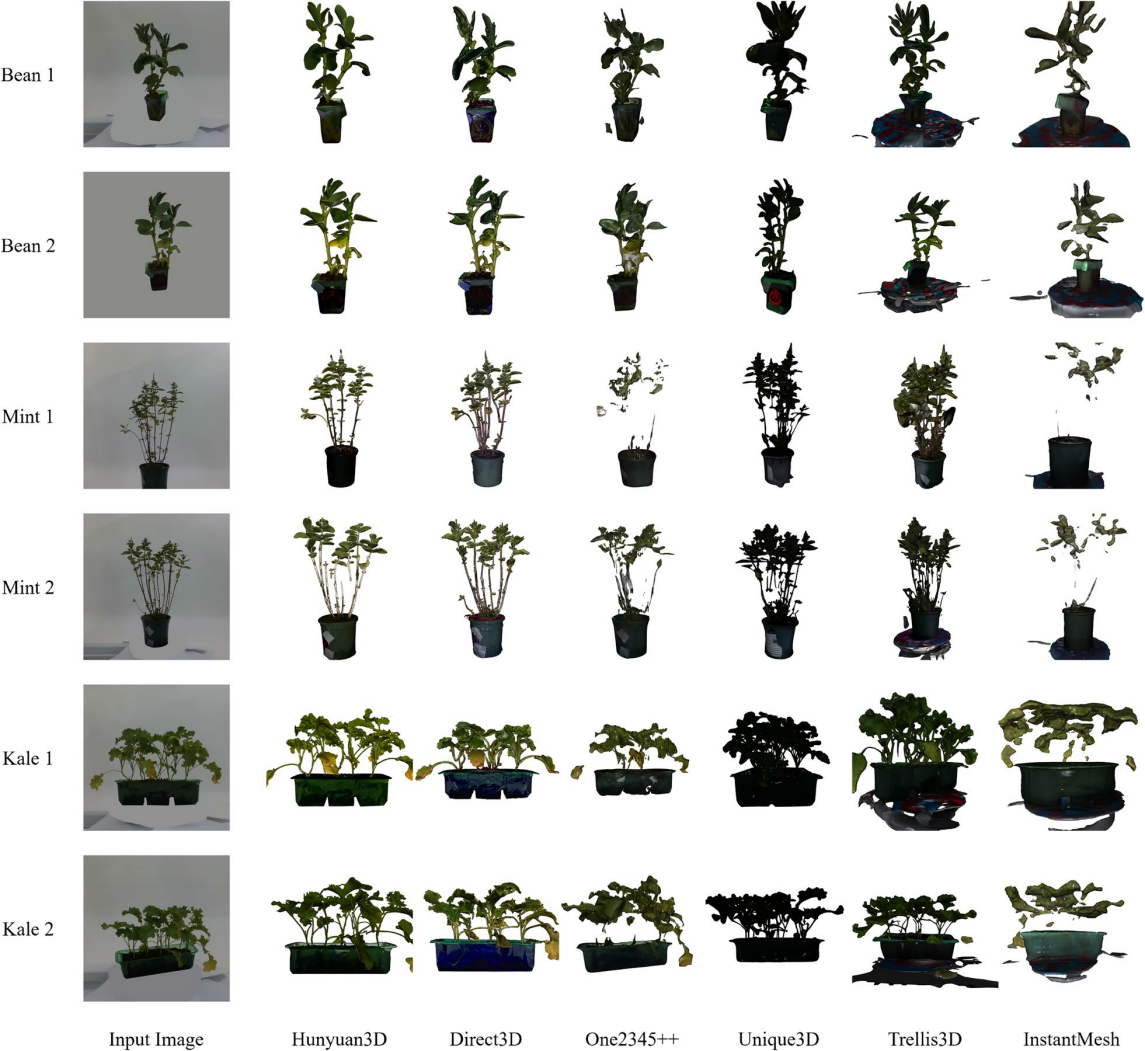
Example images of resulting models. For each species, two example instances are shown with generated meshes by all six methods

In addition to quantitative analysis, qualitative evaluations were carried out to assess visual performance and quality, and for this we use the other plants to show generalisation. Figure 5 illustrates representative examples of generated plant meshes alongside the corresponding input images for bean plant instances and kale and mint instances, visually demonstrating the differences in reconstruction quality among the methods.

Qualitative comparisons confirmed Hunyuan3D’s superior capability in capturing intricate plant details, such as accurate leaf shapes, clear vein structures, and realistic branching patterns. The generated meshes from Hunyuan3D consistently exhibited close correspondence with the ground-truth geometry, preserving fine-scale details even in complex areas. Despite occasional minor inaccuracies in spatial alignment, Hunyuan3D outputs appeared highly realistic and faithful to the input images, especially for Beans and Kale.

Trellis3D’s generated meshes were visually coherent and captured the general morphology of the plant instances effectively. Nonetheless, detailed qualitative analysis revealed limitations in fine structural accuracy, like mint examples as shown in Fig. 5. Specifically, leaf edges and delicate branching structures were sometimes oversimplified or inadequately represented. While visually acceptable at moderate resolutions, close inspections revealed lower fidelity in intricate plant regions compared to Hunyuan3D.

Meshes generated by One2345++ displayed more deficiencies. Although certain generated models appeared visually acceptable from afar, closer scrutiny revealed substantial structural irregularities, including distorted branching structures, incomplete stem and leaf geometries, and significant spatial inaccuracies as shown in Fig. 5. These visual issues corresponded directly with quantitative findings, particularly lower IoU scores, indicating difficulties in reconstructing coherent 3D plant structures.

InstantMesh reconstructions presented the greatest degree of visual abstraction and simplification among all evaluated models. Generated meshes typically lacked detailed geometric features, such as accurate leaf shapes or branching structures, resulting in generalised forms and simplified geometries. However, InstantMesh excelled in generating visually smooth and coherent surface normals, albeit at the cost of significant detail loss, aligning closely with its quantitative performance characteristics.

Direct3D and Unique3D also exhibit distinctive qualitative characteristics. Direct3D tends to generate smoother surfaces with reasonable global structure but often underestimates thin leaves and fine branching regions, especially when these are heavily occluded in the single input image. Unique3D, while producing visually coherent meshes, frequently exhibits mild over-smoothing on leaf boundaries and occasionally merges adjacent leaves into thicker structures. These observations align with their quantitative performance: both models perform competitively on global metrics but struggle with thin, high-frequency plant geometry compared to Hunyuan3D.

Overall, qualitative evaluation reinforced the quantitative findings, highlighting Hunyuan3D as the most visually accurate and semantically consistent method. Trellis3D exhibited acceptable visual results but was limited in capturing fine structural details, while InstantMesh and One2345++ presented significant qualitative shortcomings, reflecting their lower quantitative performance metrics.

## Discussions

### Summary of principal findings

This study set out to determine how well contemporary image-conditioned 3D generative models recover realistic plant geometry from a single photograph, using a unified protocol on the PlantDreamer Bean subset, further verified by qualitative evaluation on mint and kale examples. Three conclusions emerge clearly from the results. First, Hunyuan3D delivered the strongest overall accuracy and semantic agreement with the input image, as reflected by the best Chamfer Distance and F-Score and the highest adapted CLIP score, while maintaining competitive surface normal agreement. Second, Direct3D formed a robust second tier, balancing strong geometry with good visual and semantic fidelity, suggesting that direct mesh-oriented pipelines can approach the accuracy of heavier diffusion-based systems. Third, methods designed primarily for speed or single-step inference–InstantMesh and, to a lesser extent, One2345++–sacrificed geometric completeness and volumetric overlap, even when their surfaces appeared smooth or their perceptual scores were acceptable. Trellis3D occupied a middle ground: its PSNR was highest, indicating faithful view-wise appearance under this rendering protocol, but its geometry and perceptual similarity lagged behind the best models.

### Model-specific behaviour in the context of plant morphology

The quantitative and qualitative analyses aligned with the structural demands of botanical targets. Hunyuan3D’s two-stage pipeline, separating shape diffusion from texture synthesis, consistently preserved leaf outlines, stem thickness, and overall leaf arrangement, and produced meshes that visually tracked the input photograph more closely than the other systems. Nevertheless, the method exhibited occasional spatial inconsistencies when confronted with dense, self-occluding foliage of the sort this study observes more often in Mint than in Bean; this manifested as local misplacements of leaf surfaces or slight branch drift during alignment, which in turn depressed normal consistency and IoU in those cases.

Trellis3D benefited from its structured latent space in terms of global spatial coherence: reconstructions were frequently “complete plants” with plausible topology, even when fine details were softened. The method’s top PSNR suggests its renders align well at the pixel level with the ground-truth views under a fixed camera, yet its higher LPIPS implies that high-frequency perceptual details and textural micro-structure are not as faithfully captured as by Hunyuan3D or Direct3D.

One2345++ achieved reasonable Chamfer and perceptual scores but struggled most with intricate structures. Qualitatively, leaf clusters often merged, and thin elements were sometimes under-reconstructed, leading to low IoU and a relatively variable F-Score. These issues are consistent with a single-step implicit pipeline that does not explicitly regularise for thin, highly occluded botanical parts.

InstantMesh excelled in surface smoothness and produced the highest normal consistency on average, yet its geometric overlap and completeness were the weakest. This combination–good normals with poor IoU and F-Score–reflects simplified, smoothed surfaces that lack the fine, articulated geometry critical for plants. In practice, the method’s speed is compelling for interactive scenarios, but its reconstructions under-represent thin leaves and inter-leaf gaps.

### Interpreting metric disagreements on botanical targets

Several metric pairings illuminate plant-specific behaviour. The simultaneous lead of Trellis3D on PSNR versus its weaker LPIPS indicates that pixel-wise similarity under this renderer does not guarantee human-perceived fidelity for foliage textures; LPIPS, being feature-based, penalises the loss of high-frequency fine patterns and edge details that PSNR overlooks. InstantMesh’s strong normal consistency with poor IoU reiterates that smooth, well-oriented surfaces can still miss volumetric occupancy in thin regions. Across methods, absolute IoU values were low compared to typical rigid-object benchmarks. This is expected: IoU is sensitive to voxelisation and penalises thin structures disproportionately; a leaf that is one to two voxels thick in a discretised volume is easy to miss even when its surface is well aligned. These observations argue for plant-aware evaluation that complements surface metrics (Chamfer, normal consistency, F-Score) with structure-aware measures such as skeleton similarity, branch graph edit distance, or leaf-level detection/segmentation accuracy in future studies.

The adapted CLIP score, computed as cosine similarity between image embeddings of generated and ground-truth renders, correlated well with the subjective sense of “does this look like the same plant from this view?”. Unlike the usual text–image use of CLIP, the image–image variant captures semantic and perceptual agreement without privileging pixel identity. Hunyuan3D’s lead on this metric mirrors its qualitative alignment to the input photograph and supports using adapted CLIP as a practical semantic proxy when botanical annotations are scarce.

### Effects of pre-processing and alignment

**Fig. 6:**
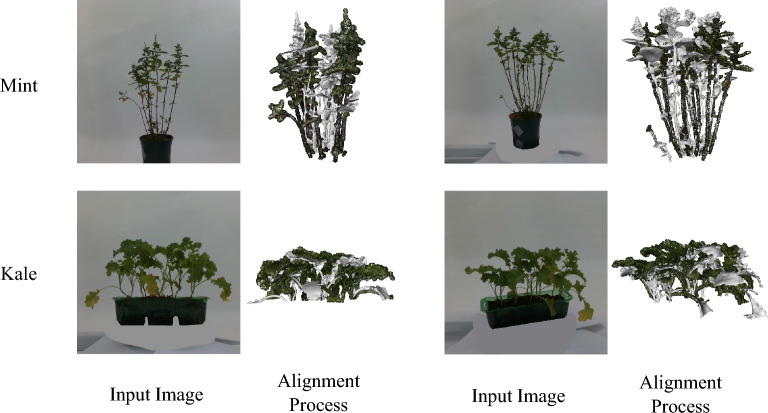
Example images of the mesh alignment for a generated mesh (grey) and corresponding ground truth (coloured) mesh reconstructed from ground truth point clouds. Mesh alignment for Mint and Kale plant needs more manual refinement due to the more complex topology and more severe self-occlusion

Accurate evaluation of plant reconstructions hinges not only on the generative model itself but also on the quality of input preparation and output alignment. Input image preparation proved consequential. When models lacked robust internal matting, feeding segmented images (background removed) to the models reduced spurious conditioning from the pot, base, and studio backdrop, and produced measurably cleaner geometry around the crown and lower stem. Pre-processed images in which the base region was painted out also helped stabilise methods sensitive to background edges. On the target side, cropping pots and bases from both generated and ground-truth meshes before scoring eliminated confounds and ensured that scores reflected plant reconstruction rather than the fidelity of ancillary objects.

Mesh alignment was a non-trivial source of variance. Semi-automatic alignment, followed by manual refinement, was adequate for most Hunyuan3D and Direct3D outputs, which tended to be close to the reference pose. Conversely, methods with larger geometric deviations, notably InstantMesh and some One2345++ reconstructions, required substantial manual intervention. Trellis3D and Unique3D generally aligned well at the global scale but often exhibited moderate local mismatches, as shown in Fig. 6, necessitating occasional manual refinements, though to a lesser degree than InstantMesh or One2345++. Because Chamfer, IoU, and F-Score are all sensitive to residual misalignment, improving registration robustness–e.g., combining global feature-based initialisation with robust ICP variants that down-weight outliers–would further reduce evaluation noise, particularly for thin structures whose local correspondences are fragile. The need for manual refinement also underscores a practical point for plant scientists: deploying single-image 3D generation in phenotyping pipelines will require either reliable automatic alignment or direct metric learning on view-rendered images that is less pose-sensitive.

Overall, these findings highlight that both input pre-processing and alignment accuracy substantially affect reconstruction quality and metric reliability. The reliance on semi-manual alignment currently limits scalability, underscoring the need for robust automatic registration methods tailored to botanical geometry, or alternatively, evaluation protocols based on view-rendered images that are inherently less pose-dependent.

### Implications for plant science applications

**Fig. 7:**
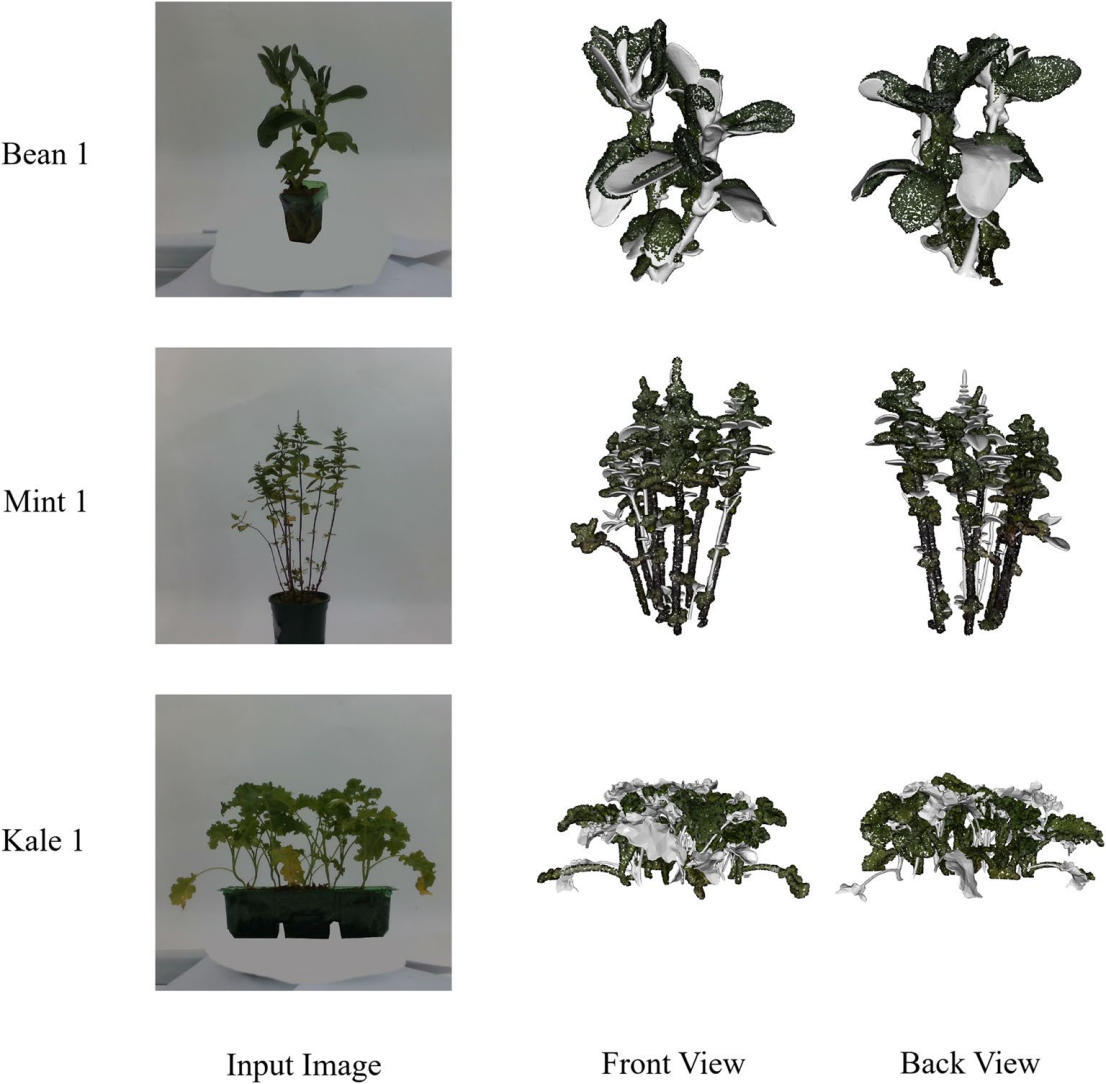
Example images of the reconstructed meshes and overlays of ground-truth (coloured) and generated (grey) models as alignment visualisation for Hunyuan3D from front and back view. The reconstructions from the front view are more precise compared to the back view. For more views of reconstruction, please refer to Extended Data Fig. 8

From the perspective of digital phenotyping and botany, method choice should align with the downstream task. Where morphological measurements demand accurate leaf boundaries, petiole thickness, and plausible global topology–e.g., leaf count, leaf area estimation, or branching order–Hunyuan3D is the most suitable among the tested models, with Direct3D a close alternative when runtime or deployment simplicity is important. For interactive visualisation, rapid prototyping, or educational AR applications where speed dominates and fine-scale geometry is secondary, InstantMesh provides a practical baseline, albeit with the recognised limitations in completeness. Trellis3D’s spatial coherence suggests potential as a backbone for pipelines that will subsequently refine details. Unique3D’s coherent global structure but lower semantic alignment indicates it could benefit from tighter image-conditioned guidance or plant-specific priors.

Limited by the lack of ground-truth biological trait annotations in the dataset, and noting producing such annotations requires a dedicated data labelling step, we did not do downstream biological trait evaluation. But to support future work, the reconstructed meshes, alignment scripts, and trait-extraction procedures are provided. Meanwhile, Fig. 7 illustrates the reconstructions from the front and back view, and overlays of ground-truth (coloured) and generated (grey) models as alignment visualisation for Hunyuan3D. It shows how the reconstruction compares from the most challenging view, where the 3D plant reconstruction is shown as a function of available information. The reconstructions from the front view are more precise compared to the back view. For more views of reconstruction, please refer to Extended Data Fig. 8.

### Limitations and opportunities for methodological improvement

There are also limitations qualifying the present conclusions. First, the main analysis was conducted on ten Bean instances; while beans capture relevant thin-structure challenges, broader botanical diversity–particularly denser crowns such as Mint and more heterogeneous phyllotaxy such as Kale–will stress generalisation. Second, the reliance on public cloud implementations introduces unknowns: some services may apply undocumented defaults, lightweight finetunes where the models could have fewer parameters, or mesh post-processing to improve stability. Although the inputs and used platforms were standardised, exact parity with open-source checkpoints cannot be guaranteed. Take Hunyuan3D for example, the official platform Hunyuan-Studio introduces a bounding box and multi-view images for additional guidance, which could improve the procedure of generation and the final mesh [45]. Third, the alignment protocol, while careful, still required manual refinement in difficult cases and remains a source of variance for thin structures. Another limitation is that all experiments in this study were conducted using images where background clutter had been removed manually. Although this was necessary to ensure a fair comparison between reconstruction pipelines, real-world agricultural and greenhouse environments rarely provide such clean backgrounds. Background leaves, soil patterns, pots, or support structures can distract the generative models and potentially degrade reconstruction accuracy. Future work should investigate background-robust preprocessing or foreground segmentation modules to enable more realistic deployment scenarios. Finally, the absolute IoU values should be interpreted cautiously given the known bias of voxel occupancy toward thick parts; the inclusion of surface-based metrics mitigates but does not eliminate this effect.

The observed error modes point to concrete research directions. Incorporating plant-aware priors–such as branch skeleton constraints, leaf blade thickness regularisation, and self-occlusion reasoning–into shape diffusion or direct mesh decoders could improve reconstruction in dense crowns. A hybrid strategy that pairs a spatially coherent backbone (e.g., SLAT-style latents) with a detail-oriented refinement module (e.g., diffusion upsampler) may combine Trellis3D’s completeness with Hunyuan3D’s detail. On the data side, multi-view conditioning–even with synthetic views generated by robust view-diffusion–should reduce the “Janus” artefacts observed in single-image pipelines. Finally, evaluation can be strengthened by adding structure-aware metrics (skeleton overlap, leaf instance matching, branch angle distributions) and by adopting alignment-invariant, render-based measures that complement surface distances. Overall, it is worth noting that plant reconstruction is a particularly challenging task for these methods, and although some do not perform well here, they may perform very well on objects with different topologies and appearances.

In summary, single-image 3D plant reconstruction has reached a point where high-fidelity geometry is achievable for moderately complex specimens under controlled input preparation, with Hunyuan3D and Direct3D currently offering the best accuracy–stability trade-off. At the same time, systematically addressing thin-structure completeness, occlusion reasoning, and alignment sensitivity will be key to translating these advances into robust tools for large-scale plant phenotyping and allied applications. A quantitative evaluation of biological traits (e.g., leaf count, leaf angle, canopy spread) is an important direction for future work, but is currently limited by the lack of trait annotations in the PlantDreamer dataset.

## Conclusions

This study systematically evaluated six state-of-the-art single-image 3D generative models on the PlantDreamer dataset under a unified protocol. By comparing methods that span diffusion pipelines, structured 3D latents, implicit decoders, and direct mesh inference, we assessed their ability to reconstruct realistic plant structures from photographs.

Three main conclusions emerged. Hunyuan3D achieved the most accurate and semantically faithful reconstructions across geometry and semantic metrics. Direct3D provided a strong balance of accuracy and efficiency, showing that lighter pipelines can rival diffusion-based systems. Faster approaches such as InstantMesh and One2345++ delivered smoother but less complete geometry, while Trellis3D and Unique3D produced coherent global structures but missed fine foliage detail. Qualitative inspection reinforced these findings and highlighted the trade-offs between accuracy, efficiency, and detail across current approaches.

For plant-science applications, Hunyuan3D is the most reliable choice when accurate morphology is required, while Direct3D offers a practical alternative when efficiency matters. InstantMesh may still be suitable for interactive or educational uses, provided its limitations are acknowledged. The limitations of this study point to future needs: incorporating plant-aware priors, hybridising spatial and detail-oriented approaches, and developing structure-aware evaluation protocols.

In sum, single-image 3D plant generation is now capable of producing high-fidelity reconstructions for moderately complex specimens under controlled input preparation. Among current options, Hunyuan3D and Direct3D offer the best trade-offs for accuracy-driven plant analysis, while faster pipelines remain attractive for interactive uses. By codifying a plant-centric protocol, highlighting error modes tied to botanical structure, and identifying metrics that better reflect plant morphology, this work provides a practical baseline and a roadmap for turning image-conditioned 3D generation into a reliable tool for digital plant phenotyping, crop science, and ecological research.

## Figures and Tables

**Fig. 8:**
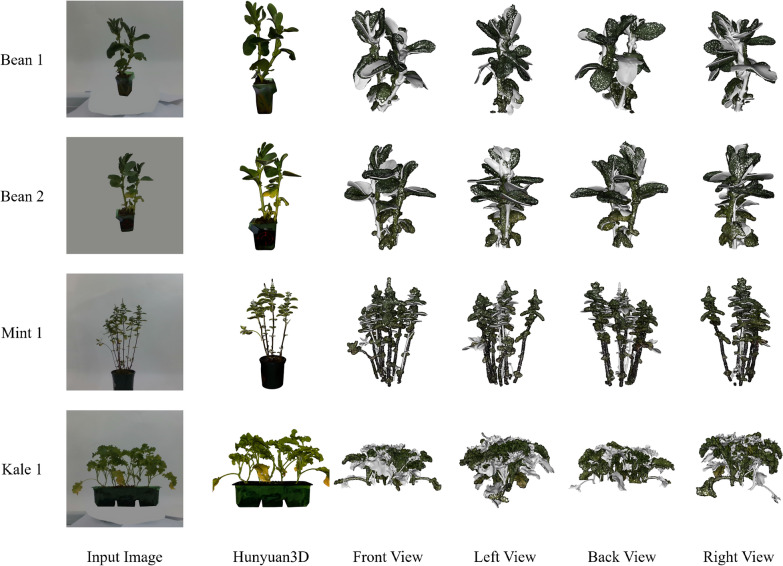
Example images of the reconstructed meshes and overlays of ground-truth (coloured) and generated (grey) models as alignment visualisation for Hunyuan3D, from 4 different views including front, left, back and right views. This figure illustrates how good the reconstruction is from the other view, where the 3D plant reconstruction is shown as a function of available information. The reconstructions from the front view are more precise compared with other views

## Data Availability

The data supporting this publication will be made available through the University of Nottingham Research Data Repository. A persistent identifier (DOI) will be included here upon acceptance: [DOI – to be updated].

## Appendix

### Extended Data A reconstructions and overlays from multi-view

See Fig 8
